# The influences of the between- and within-run components of variation on the mean rule

**DOI:** 10.1155/S1463924686000184

**Published:** 1986

**Authors:** Pierre Douville, George S. Cembrowski, Jerome F. Strauss

**Affiliations:** 1 Wm. Pepper Laboratory Department of Pathology and Laboratory Medicine Hospital of the University of Pennsylvania 3400 Spruce Street Philadelphia Pennsylvania 19104 USA; 2 L'Hotel-Dieu de Quebec City Quebec Canada


Journal of Automatic Chemistry/*Journal of Clinical Laboratory Automation*, Vol. 8, No. 2 (April–June 1986), pp. 85–88

### The influences of the between- and within-run components of variation on the mean rule

**Pierre Douville*, George S. Cembrowski† and Jerome F. Strauss**

*Wm. Pepper Laboratory, Department of Pathology and Laboratory Medicine, Hospital of the University of Pennsylvania, 3400 Spruce Street Philadelphia, Pennsylvania, 19104, USA*

### Introduction

Quality control materials are usually described by their means and 'total' standard deviation ($s_t$). Analysis of variance can be used to separate the total standard deviation into its within-run ($s_w$) and between-run ($s_b$) components [1]:

$$s_t = (s_w^2 + s_b^2)^{1/2} \quad (1)$$

Most assays performed in chemistry and endocrinology laboratories have values of $s_b$ which are non-zero with the ratio $s_b/s_w$ varying from 0.3 to 3 [2 and 3]. Westgard, Falk and Groth have recently used computer simulations to show how $s_b$ and $s_w$ affected the performance characteristics of various control rules [4 and 5]. Their simulations were based on a model that incorporated $s_b$, $s_w$ and errors of varying sizes, either systematic or random. The simulations were used to obtain the probability of false rejection ($p_{fr}$), the probability of a rejection signal when no analytical error was present; and the probability of error detection ($p_{ed}$), the probability of a rejection signal when an analytical error was present. They found that with significant $s_b$, there was deterioration in the performance characteristics of rules sensitive to systematic error, i.e. there was a tendency for $p_{fr}$ to increase and $p_{ed}$ to decrease. They concluded that the optimal detection of systematic errors was difficult in the presence of significant $s_b$.

An alternative model, which more realistically describes the interpretation of control observations by the laboratorian, is presented. Use of this model yields performance characteristics which differ significantly from the Westgard approach and demonstrates how a specific control procedure, the mean rule, can be optimized for analytical methods. The mean rule is a simple, powerful procedure for the detection of systematic errors and has been recommended in various quality control schemes [6]. Its simplicity permits direct calculation of the probability of rejection without computer simulations.

* Pierre Douville can be contacted at L'Hotel-Dieu de Quebec City, Quebec, Canada.

† Author to whom correspondence should be sent.

### Models and methods

### *Mean rule*

A mean rule, $\bar{x}_{0.01}$, refers to the control rule for which the mean of a set of $N$ measurements exceeds the control limits which give a 1% frequency of false rejections ($P_{fr}$ = 0.01) [7]. When a method is in control, the mean of the controls for each run is distributed about a grand mean $\bar{x}$ with a standard deviation of $(s_w^2/N + s_b^2)^{1/2}$ [5]. For a Gaussian distribution, the limits for the $\bar{x}_{0.01}$ rule are:

$$\text{Upper Limit} = L_U = \bar{x} + 2.58\ (s_w^2/N + s_b^2)^{1/2} \quad (2)$$

$$\text{Lower limit} = L_L = \bar{x} - 2.58\ (s_w^2/N + s_b^2)^{1/2}. \quad (3)$$

### *Westgard Model (Model 1)*

In this model, groups of control data with given means, $s_w$, $s_b$ and variable amounts of systematic error or random error are simulated and inspected for violation of particular rules or sets or rules [4]. To simulate control results, the error components, $s_b$ and $s_w$, are first added to the true mean, $\bar{x}$. Because $s_b$ and $s_w$ can either be positive or negative, the resulting control data are distributed symmetrically about $\bar{x}$. Then to simulate systematic error ($SE$), the shift $\Delta SE$ is added to the control results. The control results are then distributed about $\bar{x} + \Delta SE$. Figure 1($a$) shows the symmetrical distribution of the control observations about $\bar{x} + \Delta SE$; the standard deviation is $(s_w{}^2/N + s_b^2)^{1/2}$. The exact probabilities of rejection by the mean rule can be calculated. The control mean will be distributed about $\bar{x} + \Delta SE$ and the normalized distance between the mean and the control limits will be:

$$\text{Distance 1: } Z_U = (L_U - (\bar{x} + \Delta SE))/(s_w^2/N + s_b^2)^{1/2} \quad (4)$$

$$\text{Distance 2: } Z_L = (L_L - (\bar{x} + \Delta SE))/(s_w^2/N + s_b^2)^{1/2}. \quad (5)$$

The probability that the control mean is outside either the upper or lower control limits is equal to the sum of the probabilities that correspond to the upper and lower normalized distances (found in any Z-distribution table).

### *Alternative Model (Model 2)*

To determine the presence of systematic error in an analytical run, one or more control rules are used to compare the control results to the stable mean. In the application of the mean rule, the total shift (shift$_t$) or the difference between the stable mean and the current batch mean is monitored. The total shift is equal to the sum of any systematic error plus the shift due to the between run component. Model 2 duplicates the manner in which individual runs are reviewed in the laboratory. In this model only shift$_t$ is added to the true mean. As shown in

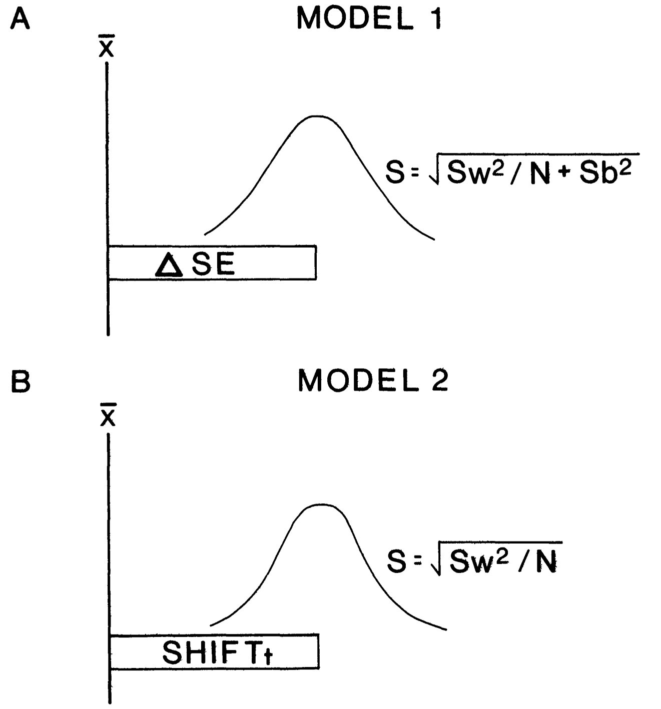

*Figure 1. Comparison of the location and distribution of the means of N control observations. Figure 1(a) shows Model 1; figure 1(b) shows Model 2.*

figure 1(*b*), the mean of controls is distributed about $\bar{x}$ + shift$_t$. Within a run, $s_w$ is the only source of random error and the normalized distance between the new mean and the upper and lower control limits become:

$$\text{Distance 1: } Z_U = (L_U - (\bar{x} + \text{shift}_t))/(s_w^2/N)^{1/2} \quad (6)$$

$$\text{Distance 2: } Z_L = (L_L - (\bar{x} + \text{shift}_t))/(s_w^2/N)^{1/2} \quad (7)$$

The probabilities that the new mean is outside either the upper or lower control limit can be calculated as for Model 1.

### Methods

For Model 1, the systematic error was varied from 0 to 5 $s_t$ in multiples of 0.5 $s_t$ for the following values of $s_b/sw$: 0, .5, 1, 2, and infinity ($s_w$ = 0). The probability of the mean of one and four observations exceeding its control limits was calculated with equations (4) and (5). The probability was then plotted against the size of the *systematic error*, expressed in multiples of $s_t$.

For Model 2, the total shift was varied from 0 to 5 $s_t$ in multiples of 0.5 $s_t$ for the following values of $s_b/s_w$: 0, .5, 1, 2, and infinity ($s_w$ = 0). The probability of the mean of one and four observations exceeding its control limits was calculated with equations (6) and (7). The probability was then plotted against the size of the *total error* expressed in multiples of $s_t$.

To illustrate the differences between Model 1 and Model 2, power functions were generated as described above for $s_b/s_w$ = 1 and $N$ = 1, 2, 4 and 8.

### Results

Power function curves for the mean rule for Models 1 and 2 are shown in figures 2 and 3, respectively. Figure 2 (Model 1) shows the performance characteristics of the mean rule for the detection of systematic error expressed in multiples of $s_t$. With one control the probability of detecting a systematic error of 2 $s_t$ is 0.28, regardless of the ratio, $s_b/s_w$. However, with four controls and increasing $s_b/s_w$, there is a marked reduction of efficiency. For example, the probability of detecting a systematic error of 2 $s_t$ decreases from 0.92 to 0.28 as $s_b$ increases from 0 to $s_t$.

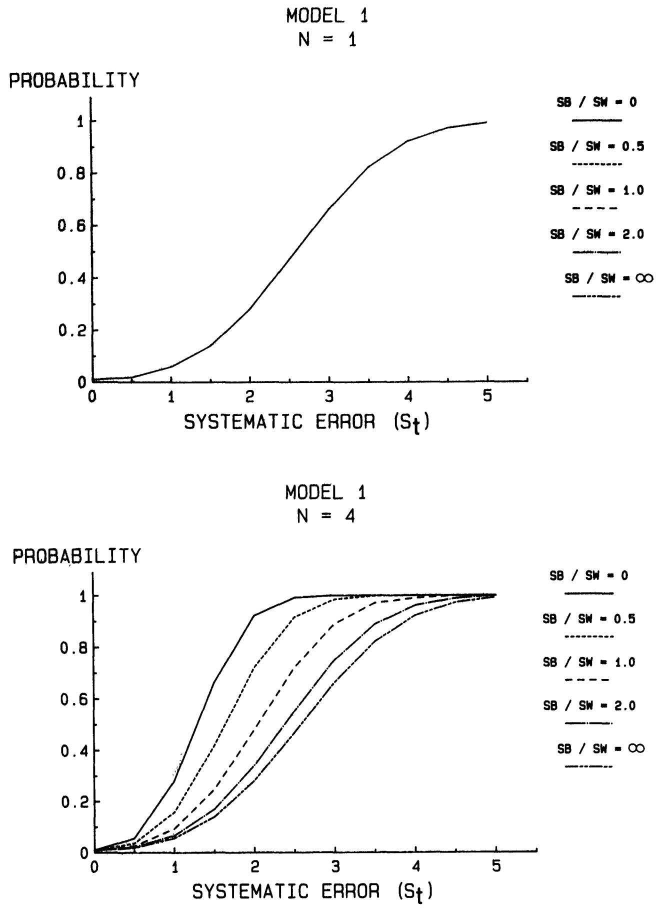

*Figure 2. Power function curves for the $\bar{x}_{0.01}$ rule using Model 1 for $N$ = 1 (figure 2[a]) and $N$ = 4 (figure 2[b]). The ratio, $s_b/s_w$, is varied from 0 to ∞.*

In figure 3 (Model 2) the scale on the abscissa is also expressed as multiples of $s_t$. When $s_b$ = 0 ($s_b/s_w$ = 0), the Model 2 power functions are identical to those of Model 1. For Model 2, the ratio $s_b/s_w$ affects the curves even for $N$ = 1. As $s_b/s_w$ increases, small shifts are detected less frequently and large shifts more frequently. For $N$ = 4 the curves are steeper and are displaced to the right in proportion to the importance of $s_b$. For $s_b/s_w$ approaching infinity the power functions approximate a step function and are not influenced by $N$.

Figure 4 shows the effect of averaging different numbers of controls for both models. As $N$ increases, both models

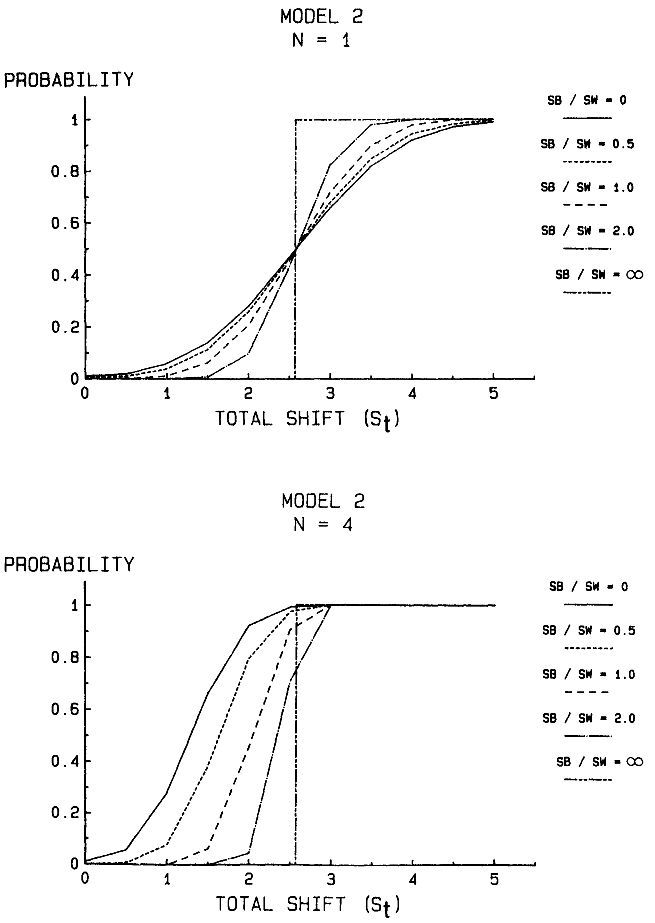

*Figure 3. Power function curves for the $\bar{x}_{0\cdot01}$ rule using Model 2 for $N = 1$ (figure 3[a]) and $N = 4$ (figure 3[b]). The ratio $s_b/s_w$ is varied from 0 to $\infty$.*

show improved performance. For model 2 and large $N$, the curve approaches a step function and indicates certainty about the presence and magnitude of the shift.

### Discussion

In Model 1, for each run generated, the total systematic deviation from the true mean is given by $\Delta SE$ plus the contribution given by $s_b$. Since the contribution of $s_b$ can be either positive or negative, the total deviation can be greater or smaller than $\Delta SE$. For all the runs generated, the mean deviation is thus $\Delta SE$. $\Delta SE$ may be thought to represent a long-term error that occurs over many runs, while $s_b$ represents the short-term shifts occurring from run to run. In Model 1, an effort is made to separate the contribution of $s_b$ from that of $\Delta SE$; $s_b$ becomes a source of noise that impairs the detection of the long-term systematic error $\Delta SE$. The distinction between $\Delta SE$ and $s_b$ explains the general degradation in performance for rules used to detect systematic error ($\Delta SE$) with increasing $s_b/s_w$. The laboratorian does not distinguish, however, between the long-term and short-term shifts at the end of an analytical run. He is interested in their total effect. These considerations prompted the adoption of Model 2 in which only two components are required to describe a run, the systematic component, expressed by shift$_t$ and the random component by $s_w$.

The power function plots of the mean rule derived from Model 2 for nonzero $s_b$ differ significantly from the Model 1 plots, both for $p_{fr}$ and $p_{ed}$. The $p_{fr}$ of the mean rule, $\bar{x}_{0\cdot01}$, is fixed at 0·01 for both models. In Model 1, $p_{fr}$ is represented by the $y$ intercept. For Model 2, $s_b$ represents the standard deviation of shift$_t$ for a series of acceptable runs in which no systematic error is present. Approximately 95% of these acceptable runs will have a shift$_t$ below two times $s_b$. By transforming this two times $s_b$ to a corresponding multiple of $s_t$, a zone of low shift$_t$ can be defined which includes 95% of these acceptable runs. The probability of rejection should be low in this zone. The runs with a very low shift$_t$ will have a probability of rejection far below 0·01 for the $x_{0\cdot01}$ rule. Acceptable runs with a shift close to twice $s_b$ will have a probability of rejection higher than 0·01. Overall, for the $\bar{x}_{0\cdot01}$ rule, the average $p_{fr}$ for the acceptable runs will be 0·01.

Two observations emerge regarding the detection of large shifts. First, for high $s_b/s_w$, the probability of rejection changes abruptly, from a very low level to a high level as shift$_t$ increases. This reflects a high certainty about the size of the shift when the noise produced by $s_w$ is low. The power function curves do not change significantly with replicate analysis. Second, with low $s_b/s_w$, the increased

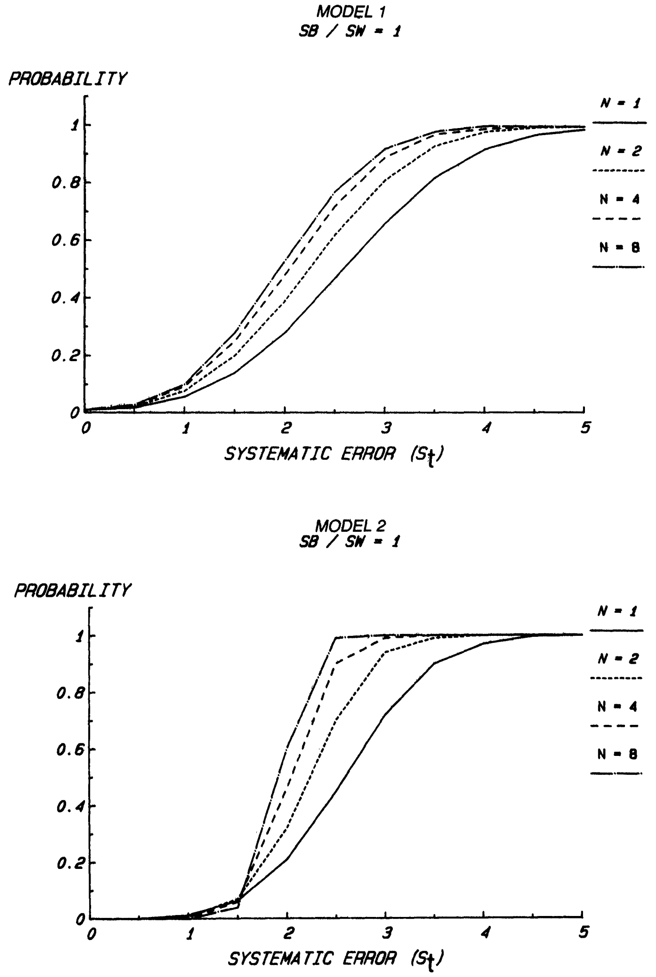

*Figure 4. Power function curves for the $\bar{x}_{0\cdot01}$ rule for Model 1 (figure 4[a]) and Model 2 (figure 4[b]) with $s_b/s_w = 1$ and $N = 1, 2, 4$ and 8.*

$p_{ed}$ with increasing shift$_t$ is more gradual and reflects the noise produced by $s_w$. Replicates improve the efficiency markedly and allow detection of even small shift$_t$.

Use of the mean rule with actual laboratory data yields a low $p_{fr}$ for 'in control' runs and a high $p_{ed}$ with significant total shifts. The creatinine method, for example, on the Hitachi 705 has been shown to have ratios of $s_b/s_w$ ranging from 0.5 to 1 depending on the concentration of the control [2]. For a ratio $s_b/s_w$ of 1, $s_b = s_t/\sqrt{2}$—equation (1). A shift$_t$ of $2s_b$ is equivalent to 1·4 $s_t$ and implies that approximately 95% of the 'in control' runs have a shift$_t$ below 1·4 $s_t$. As expected, figure 4(*b*) indicates a low probability of rejection for shift$_t$ below 1·4 $s_t$, because the mean rule with a low $p_{fr}$ was used. The probability of detecting a significant shift increases greatly with increased numbers of observations. Figure 4(*b*) shows that for a total shift of 2·5 $s_t$, the probability increases from 0·45 ($N = 1$) to 0·99 ($N = 8$).

Model 2 indicates that the performance of the mean rule is not degraded with $s_b$. On the contrary, only one or two controls are required to detect shifts larger than usual when the ratio $s_b/s_w$ is high. A high $s_b/s_w$ must not be perceived as ideal as it only reflects the fact that large shifts are accepted as part of the normal variation. The ideal situation, in which no significant shift ($s_b/s_w$ is low) occurs between runs, necessitates meticulous attention, to reduce the between-run sources of errors, for example calibration errors. In addition, as shown in figure 3, many control measurements are necessary to obtain the desired probability of error detection in order to maintain the performance. In practice, there is often a trade-off between the effort for control and the size of the tolerated shifts.

While the mean rule can be a powerful tool for the detection of systematic deviation, its use is not always practical. For most instruments, two different levels of control material are analysed per batch. Averaging the normalized values of each level may not be acceptable because the systematic shifts might be different at each level. In this situation, a multi-rule approach is probably more appropriate [9]. However, long-term averages of successive values of control material can easily be computed. Cembrowski *et al.* described an application of the mean of controls that permits an accuracy trend analysis by the continuous monitoring of the mean [10]. We think that this accuracy trend analysis will increase the usefulness of the information provided by reference samples. Moreover, because of its excellent performance characteristics for the detection of systematic shifts, the mean should be used to analyse patient data [8].

### References

1. Krouwer, J. S. and Rabinowitz, R., *Clinical Chemistry*, **30** (1984), 290.

2. Douville, P. and Forest, J. C, *Clinical Chemistry*, **29** (1983), 692.

3. Cembrowski, G. S., Douville, P. and Strauss, J. F., manuscript in preparation.

4. Westgard, J. O., Falk, H., and Groth, T., *Clinical Chemistry*, **25** (1979), 394.

5. Westgard, J. O. and Groth, T., *Clinical Chemistry*, **27** (1981), 1536.

6. Hainline, Adrian, Jr In: *Selected Methods for the Small Clinical Chemistry Laboratory* (American Association of Clinical Chemistry, 1982), 17.

7. Westgard, J. O., Groth, T. and Aronsson, T. *et al*, *Clinical Chemistry*, **23** (1977), 1857.

8. Cembrowski, G. S., Chandler, E. P. and Westgard, J. O., *American Journal of Clinical Pathology*, **81** (1984), 492.

9. Westgard, J. O., Barry, P. L., Hunt, M. and Groth, T., *Clinical Chemistry*, **27** (1981), 493.

10 Cembrowski, G. S., Westgard, J. O., Eggert, A. A. and Toren, E. C., *Clinical Chemistry*, **21** (1975), 1396.

### JOURNAL OF MICROENCAPSULATION

This international journal is devoted to the preparation, properties and uses of individually encapsulated small particles. Its scope extends beyond microcapsules to all other small particulate dosage forms which involve preparative manipulation. These forms find a wide variety of medical, biological, industrial and research applications. The journal covers the chemistry of encapsulating materials; the physics of such matters as release through the capsule wall; the technique of preparation of the microcapsules; its content and storage; and the many uses to which microcapsules are put. An important feature of *Journal of Microencapsulation* is a substantial PATENT BRIEFING and LITERATURE ALERTS section.

*Subscription enquiries to Taylor & Francis; editorial queries to Dr J. R. Nixon, Chelsea Department of Pharmacy, King's College, London, Manresa Road, London SW3 6LX.*

